# Supplementary material for: Excellent Catalytic Performance of ISOBAM Stabilized Co/Fe Colloidal Catalysts toward KBH4 Hydrolysis
Source: Nanomaterials (Basel). 2022 Aug 30;12(17):2998. doi: 10.3390/nano12172998 (PMC9458076; doi:10.3390/nano12172998)
Supplement: Supplementary file 1 [file nanomaterials-12-02998-s001.zip › nanomaterials-1861296-supplementary.pdf]

# Supporting Information

**Excellent catalytic performance of ISOBAM stabilized Co/Fe colloidal catalysts toward $KBH_4$ hydrolysis**

Keke Guan [1,†], Qing Zhu [1,†], Zhong Huang [1,*], Zhenxia Huang [1,2], Haijun Zhang [1,*], Junkai Wang [1,2], Quanli Jia [3] and Shaowei Zhang [4]

[1] The State Key Laboratory of Refractories and Metallurgy, Wuhan University of Science and Technology, Wuhan 430081, P. R. China

[2] School of Materials Science and Engineering, Henan Polytechnic University, Jiaozuo 454003, Henan, P.R. China

[3] Henan Key Laboratory of High Temperature Functional Ceramics, Zhengzhou University, Zhengzhou 450052, China

[4] College of Engineering, Mathematics and Physical Sciences, University of Exeter, Exeter EX4 4QF, United Kingdom

[†] These two authors contributed equally to this work.

* Correspondence: huangzhong@wust.edu.cn (Z.H.);
zhanghaijun@wust.edu.cn (H.Z.)

**Table S1** Batch compositions and processing conditions for the preparation of ISOBAM-104 stabilized Co/Fe colloidal catalysts.

| molar ratio of ISOBAM-104 to ion concentration | molar concentration of ISOBAM-104 (mM) | metal ion concentration (mM) | chemical composition (atom ratio) |
|---|---|---|---|
| 10 | 9 | 0.9 | $Co_{50}Fe_{50}$ |
| 30 | 27 | | |
| 50 | 45 | | |
| 80 | 72 | | |
| 50 | 30 | 0.6 | |
| | 45 | 0.9 | |
| | 60 | 1.2 | |
| | 75 | 1.5 | |
| | 60 | 1.2 | Fe |
| | | | $Co_{10}Fe_{90}$ |
| | | | $Co_{30}Fe_{70}$ |
| | | | $Co_{50}Fe_{50}$ |
| | | | $Co_{70}Fe_{30}$ |
| | | | $Co_{90}Fe_{10}$ |
| | | | Co |

**Table S2** Lattice spacing and indexed reflection planes of Co and Fe.

| Faces / Element | (200) | (220) | (222) |
|---|---|---|---|
| Co: ICDD 00-015-0806 (nm) | 0.1772 | 0.1253 | 0.1023 |
| Fe: ICDD 00-006-0696 (nm) | 0.1433 | 0.1013 | 0.0828 |

**Table S3** Lattice spacing and indexed reflection planes of Co/Fe colloidal catalysts determined by HRTEM.

| Co/Fe nanoparticle | Lattice spacing (nm) | Comparison | Indexed reflection planes |
|---|---|---|---|
| 1 | 0.168 | Between Co(200) and Fe(200) 0.1772>0.168>0.1433 | (200) |
| 2 | 0.172 | Between Co(200) and Fe(200) 0.1772>0.172>0.1433 | (200) |
| 3 | 0.174 | Between Co(200) and Fe(200) 0.1772>0.174>0.1433 | (200) |
| 4 | 0.169 | Between Co(200) and Fe(200) 0.1772>0.169>0.1433 | (200) |

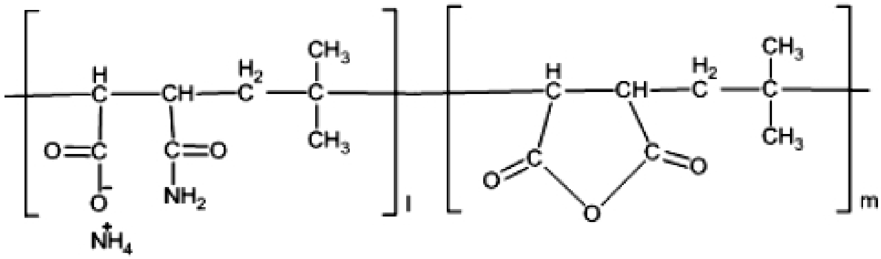

**Figure S1** The chemical structure of ISOBAM-104.

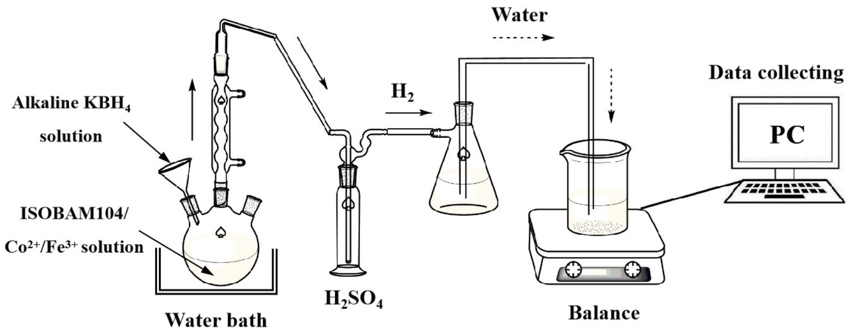


**Figure S2** The schematic diagram of experimental device for $KBH_4$ hydrolysis reaction.

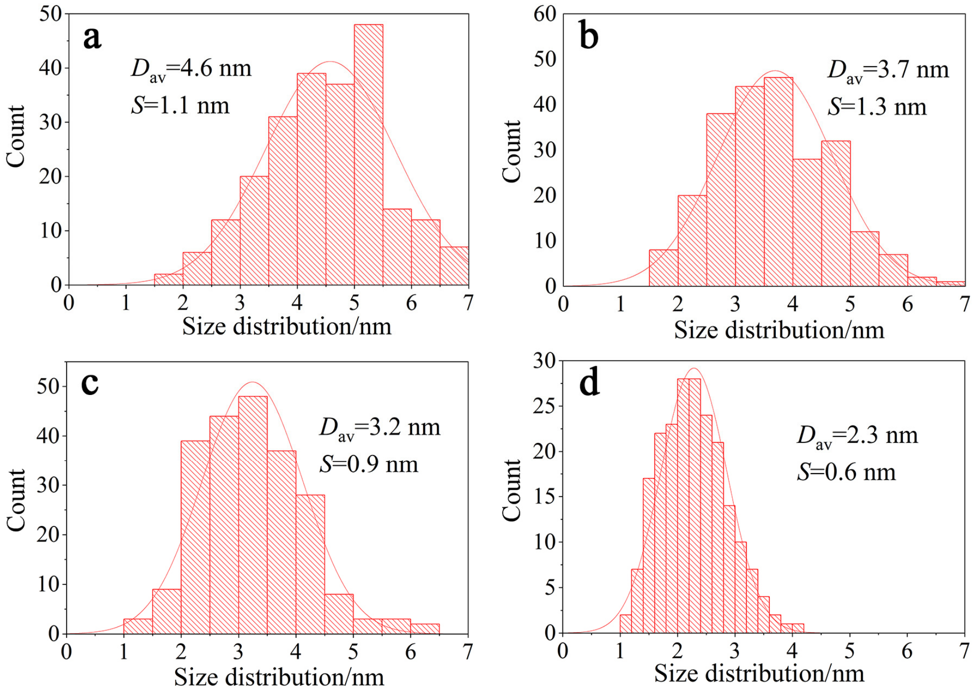


($D_{av}$: average particle size; S: standard deviation)

**Figure S3** Particles size distribution histograms of $Co_{50}Fe_{50}$ colloidal catalysts with various $R_{ISO}$ ([$Co^{2+}$+$Fe^{3+}$] = 0.9 mM; $R_{ISO}$ = 10 (a), 30 (b), 50 (c), and 80 (d)).

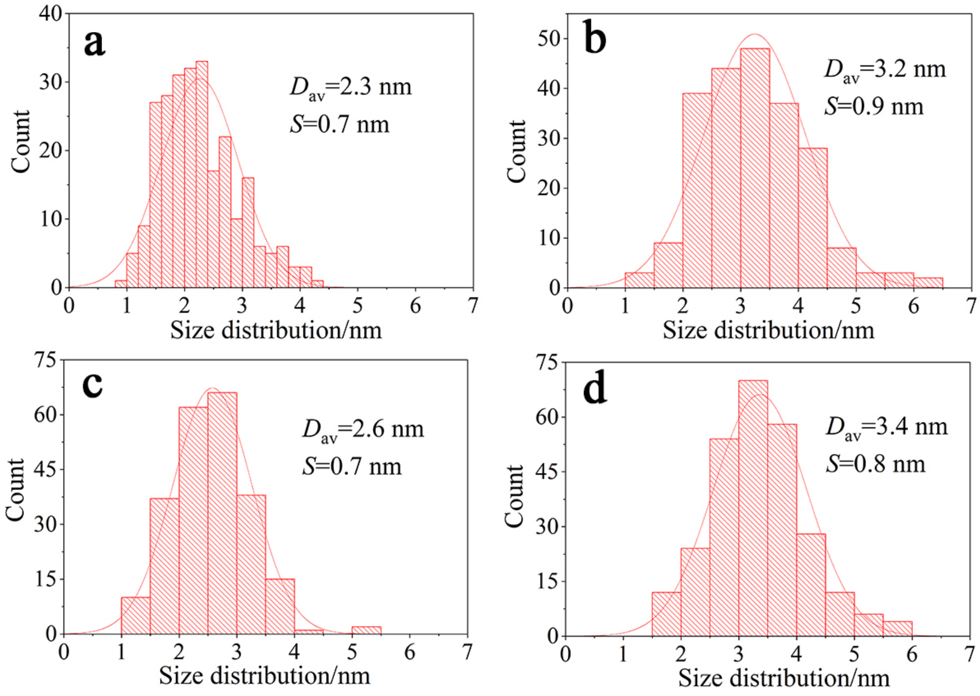


($D_{av}$: average particle size; S: standard deviation)

**Figure S4** Particles size distribution histograms of $Co_{50}Fe_{50}$ colloidal catalysts synthesized with various ion concentrations ($R_{ISO}$ = 50; [$Co^{2+}$+$Fe^{3+}$] = 0.6 (a), 0.9 (b), 1.2 (c), and 1.5 (d) mM).

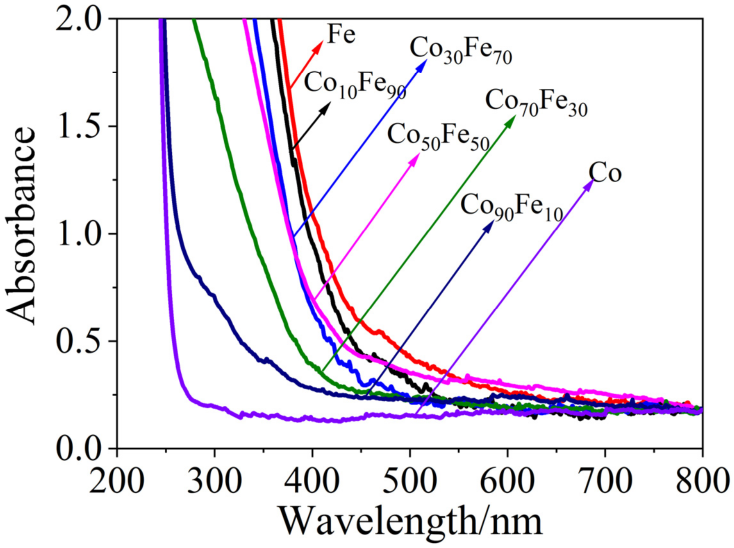


**Figure S5** UV–*vis* spectra of Co/Fe BNPs, Co and Fe nanoparticles.

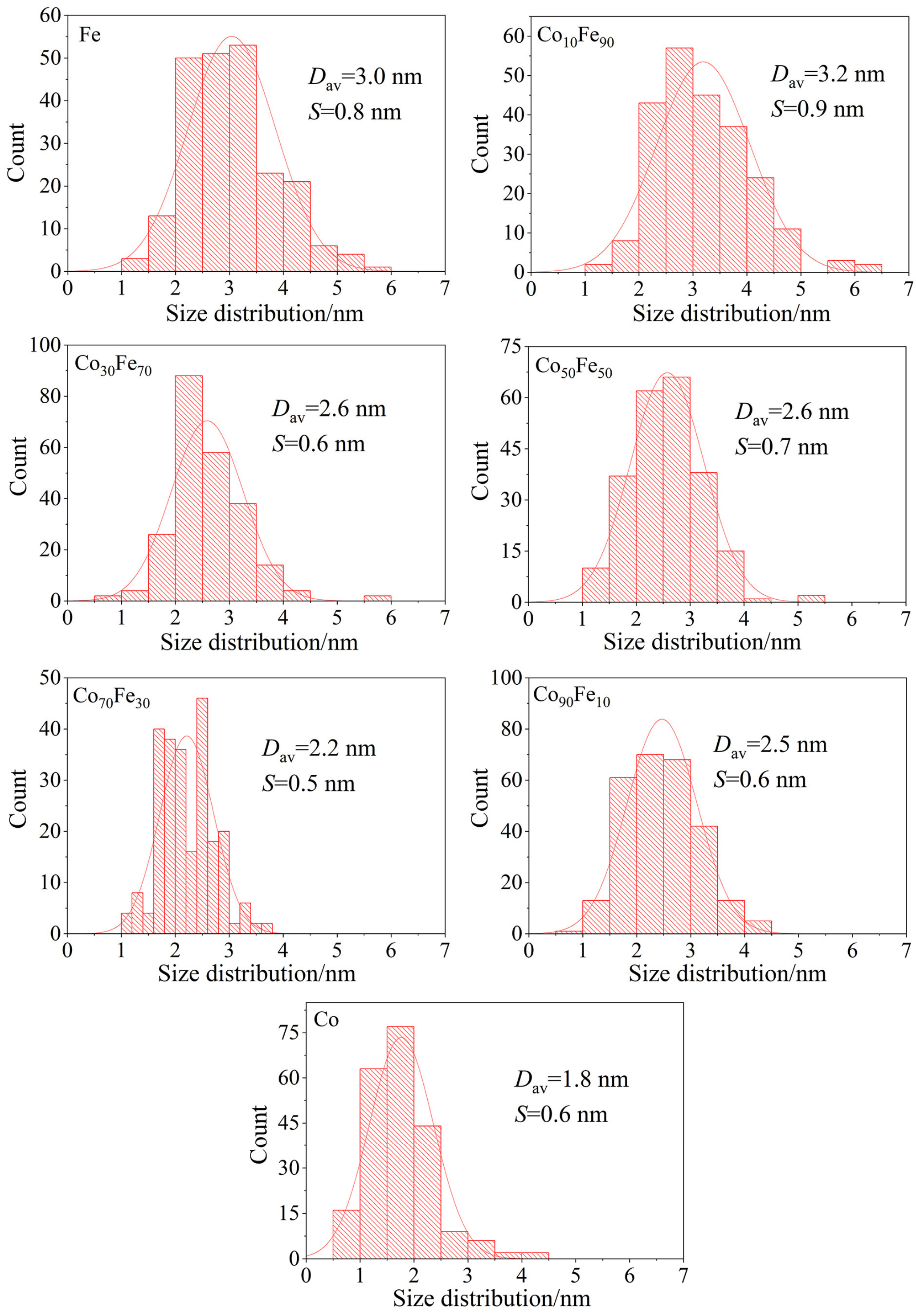


($D_{av}$: average particle size; S: standard deviation)

**Figure S6** Particles size distribution histograms of Co/Fe colloidal catalysts synthesized with various chemical compositions ($R_{ISO}$ = 50, [$Co^{2+}$+$Fe^{3+}$] = 1.2 mM).

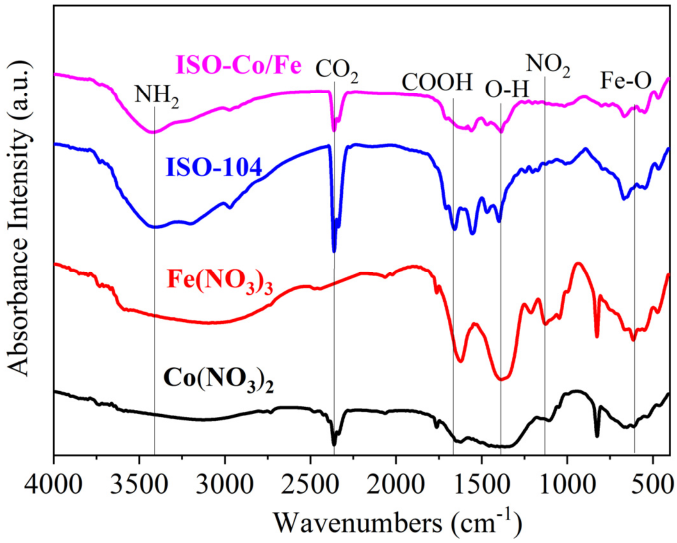


**Figure S7** FTIR spectra of ISOBAM-104, $Co(NO_3)_2$, $Fe(NO_3)_3$, and $Co_{10}Fe_{90}$ colloidal catalysts.

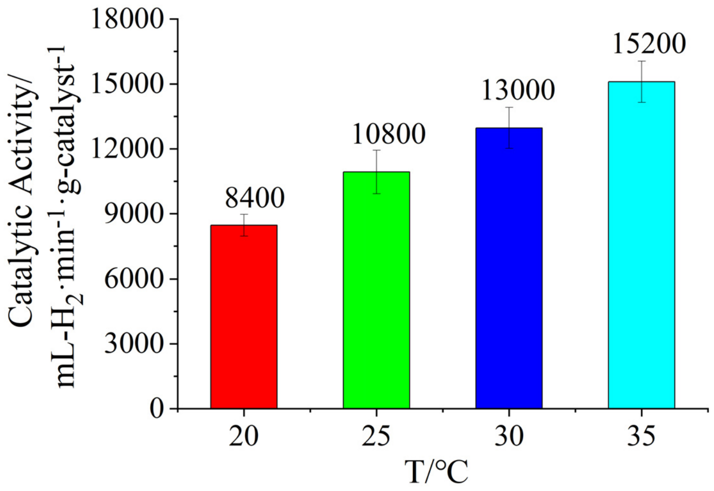


**Figure S8** Effect of temperature on the catalytic performance of $Co_{10}Fe_{90}$ colloidal catalysts.

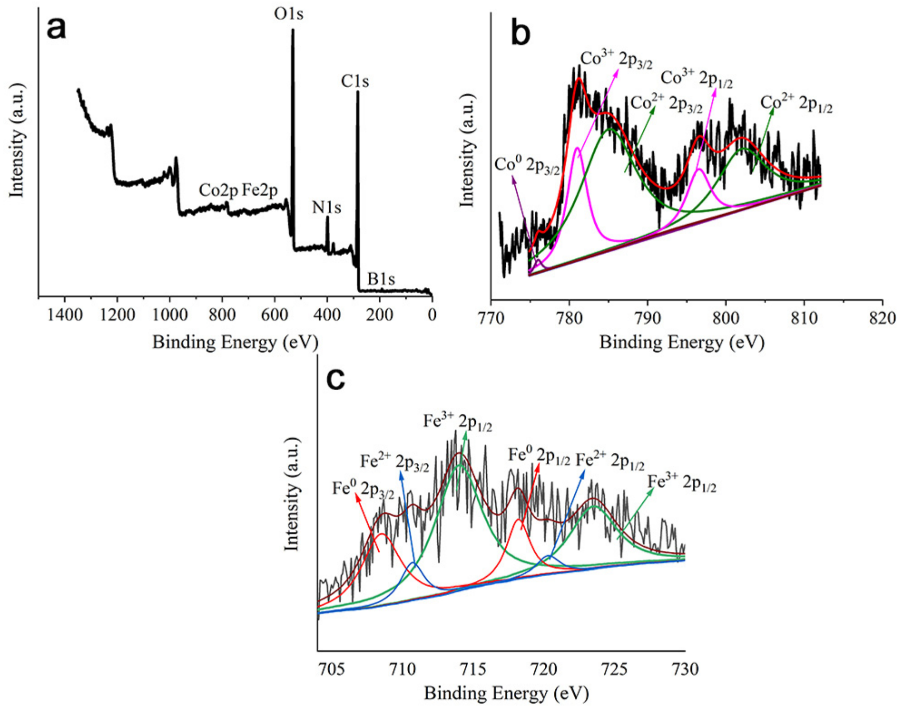


**Figure S9** XPS spectra of $Co_{30}/Fe_{70}$ colloidal catalysts: (a) total spectra, (b) Co 2p, and (c) Fe 2p.

## Calculation of catalytic activity

The catalytic activity ( $a$ , mL-$H_2$·$min^{-1}$·g-$catalyst^{-1}$) of the obtained Co/Fe colloidal catalysts can be calculated by the ratio of the hydrogen generation rate ($k$) to the mass of catalyst ($m$), as the following Equation (1):

$$a = \frac{k}{m_{cat.}} \tag{1}$$

where a represents the catalytic activity of catalysts, $k$ is the hydrogen generation rate (mL-$H_2$·$min^{-1}$), $m_{cat.}$ represents the mass of catalysts. It should be noted that the ISOBAM-104 used in this work contains $NH_4^+$ group, which also possesses a catalytic effect for $KBH_4$ hydrolysis. Therefore, under the same condition, the catalytic activities of ISOBAM-104 stabilized Co/Fe colloidal catalysts and ISOBAM-104 ($NH_4^+$ group) were respectively measured. The intrinsic catalytic activity value of Co/Fe colloidal catalysts were obtained by subtracting the value of ISOBAM-104 from that of ISOBAM-104 stabilized Co/Fe, as the following Equation

(2):

$$a_{(NPs)} = a_{(NPs/ISOBAM)} - a_{(ISOBAM)} \quad (2)$$

where $a_{(NPs)}$ represents the catalytic activity of Co/Fe catalysts, $a_{(NPs/ISOBAM)}$ is the catalytic activity of ISOBAM-104 stabilized Co/Fe colloidal catalysts, $a_{(ISOBAM)}$ represents the catalytic activity of ISOBAM-104. All the catalytic experiments were repeated no less three times under the identical condition, and the average values of the measured data were used to determine the catalytic activity. Finally, the activity values were normalized to mL-$H_2$ $min^{-1}$ g-$Co^{-1}$.based on the following Equation (3):

$$a_{Co} = \frac{a_{(Co/Fe)} - a_{Fe} * x}{1 - x} \quad (3)$$

where $a_{Co}$ represents the catalytic activity of Co atoms in Co/Fe catalysts, $a_{(Co/Fe)}$ is the catalytic activity of Co/Fe colloidal catalysts, $a_{Fe}$ represents the catalytic activity of Co atoms in Co/Fe catalysts, $x$ is the content of Fe in Co/Fe colloidal catalysts, and $1-x$ represents the content of Co in Co/Fe colloidal catalysts.
